# Vacancy in shrinking downtowns: a comparative study of Québec, Ontario, and New England

**DOI:** 10.1007/s10901-017-9587-9

**Published:** 2018-01-25

**Authors:** Justin B. Hollander, Maxwell D. Hartt, Andrew Wiley, Shannon Vavra

**Affiliations:** 1 Tufts University, Medford, MA USA; 2 Cardiff University, Cardiff, United Kingdom

**Keywords:** Shrinking cities, Vacancy, Downtown

## Abstract

In North America and around the globe, there has been emerging recognition of the size and scope of urban shrinkage, yet little is understood about how decline impacts commercial centers and downtowns. In order to facilitate geographically targeted policymaking, this paper examines the physical patterns of downtown decline in three distinct regions. We seek to test the hypothesis that differences in the process of urban decline in downtown districts vary due to national or historic context. Using statistical analysis and direct observations, we found that while the scale of population decline was greatest in New England, downtowns in both Ontario and Québec have seen substantial decline and have appeared to have better weathered the change with respect to physical signs of decline.

## Introduction

Urban decline, defined as the process of population shrinkage and property abandonment, has become an increasingly pressing issue as policymakers seek to avoid the misapplication of critical tax revenues and address broader community stabilization goals (Davey 2013). This has not gone unrecognized by the academic community, as illustrated by the formation of the Shrinking Cities International Research Network (SCIRN) (Wiechmann 2008). Evidence emerging from this—and earlier—academic inquiry suggests that building abandonment is an indicator of contemporary economic decline as well as a harbinger of future decline (Brady 1983; Accordino and Johnson 2000; Runfola and Hankins 2010; Dewar and Thomas 2013; Pallagst et al. 2014). While earlier research has contributed much to understanding the phenomenon of urban decline, less is understood about how decline impacts commercial centers and downtowns. In order to facilitate geographically targeted policymaking, this paper employs a combination of quantitative research methodologies to examine overall patterns of decline and downtown change in Québec and Ontario, Canada, and New England (Connecticut, Maine, Massachusetts, New Hampshire, Rhode Island and Vermont), USA. Comparing US and Canadian regions is an especially fruitful line of inquiry. The USA and Canada share many economic, political, and cultural traditions, but they differ as well. The neighboring regions of Québec, Ontario, and New England are major economic and trading partners, sharing liberal democratic governance frameworks, yet urban policy is much more aggressive in Canada (Haddow 2015). As a result, shrinking cities have substantially more vacancy and abandonment in US cities than Canadian (Hackworth 2016). With global attention to the resurgence of downtowns, key questions remain about how these shrinkage dynamics play out in downtowns, where poverty has tended to colocate in US cities (Silverman et al. 2016). For this paper, we seek to test the hypothesis that *differences in the process of urban decline in downtown districts vary due to national or historic context.*

## Literature review

### The phenomenon of shrinking cities and neighborhood change

Decline or shrinkage in the urban planning literature has often been used to describe population loss, employment loss, or decrease in neighborhood quality (Bradbury et al. 1982; Berg et al. 1982; Clark 1989; Rusk 1995). In the USA, dozens of cities have succumbed to massive depopulation in recent years (Hartt 2017b). According to the US Census, the City of Detroit lost 25% of its population from 2000 to 2010. But this trend is no longer confined to post-industrial Rust Belt cities: Some of the most prominent population losses in this recent period can be found in Sun Belt locales that exploded in population in the 1990s and early 2000s, including cities such as Las Vegas, Atlanta, Modesto, and Phoenix (Hollander 2011). In Canada, one of the fastest growing OECD countries, growth is predominantly limited to the major cities. Between 2006 and 2011, 46% of cities grew slower than the national average and 19% of cities lost population. No single rationale or explanation exists as to why these and other cities have declined. Shrinkage has been explained by everything from natural disasters (Vale and Campanella 2005), deindustrialization (Bluestone and Harrison 1982), suburbanization (Jackson 1985; Clark 1989), globalization (Sassen 1991; Hall 1997), and the boom-and-bust economic cycles (Rust 1975). Many shrinking cities scholars have taken a more holistic view, positing that urban growth and decline are a global multifaceted phenomenon, deeply related to broader metropolitan and global processes that include demographic change, economic restructuring, deindustrialization, and suburbanization (Oswalt and Rieniets 2006, Audirac et al. 2012; Martinez-Fernandez et al. 2012a, b; Wiechmann and Pallagst 2012; Haase et al. 2013). Although shrinking cities research has grown considerably in the past decade, the current literature lacks robust empirical cross-national studies comparing the processes of how places change and exploring the role of different institutional and regulatory frameworks.

### The landscape of shrinking cities

When individual people or groups leave an urban region, the physical form of the city does not naturally shrink—abandoned properties and infrastructure remain until it is either occupied by new tenants or maintained by a new group (i.e., see Runfola and Hankins 2010). This can have a wide variety of frequently negative feedback effects on local economies and future abandonment. Glaeser and Gyourko (2005) studied the durability of housing in their time-series sample of 321 US cities and towns with at least 30,000 residents in 1970, showing how housing prices declined at a faster rate in depopulating cities than prices grew in growing cities. Additional research has shown the role abandoned structures serve as havens for criminal activity, further deflating economic and social incentives for individuals to stay in (or move in) to an area (Wallace 1989; Wilson and Kelling, 1982).

Older cities also tend to have older buildings, with central, historic zones like downtowns tending to have some of the oldest building stock in a city (Silverman et al. 2016). Due to the advanced age of this downtown building stock, historic preservation costs may prohibit retrofits or updates allowing slum landlords to concentrate in these areas (Burchell and Listokin 1981).

Amplifying the challenges posed by urban decline, poorer economic conditions can result in lower demand for housing and, subsequently, a lower economic class of owners or renters (Hoyt 1933; Temkin and Rohe 1996). Ultimately, when demand sinks to a certain threshold level, owners tend to abandon their structures—resulting in even poorer economic conditions (Keenan et al. 1999). This process further reduces the tax base available to local municipal systems, potentially increasing crime rates and reducing funding for municipal activities such as park and road maintenance (Dewar and Thomas 2013).

Rust’s (1975) study of population and employment decline in 30 US metropolitan areas from the 1800s to the 1970s revealed much about *how* places decline. He found that these shrinking places experienced dramatic population loss and then “a long period of profound resistance to demographic or economic change which continues until the people, artifacts, and institutions which were assembled in the truncated growth era gradually erode away” (p. 169). The very physical fabric of neighborhoods—these artifacts—is expected to “erode away” in a period of decline. For many of the cases Rust studied, once the decline began, the effects are “expected to be felt most strongly a generation after the cessation of growth and to persist for up to 50 years” (p. 187). For the booming cities of today, these results present a clarion call for a new way to do planning; a new model is needed to think about neighborhood change in the face of ongoing population and housing loss (Schilling and Logan 2008; Hollander et al. 2009).

### Canadian planning and comparative context

US and Canadian planning environments have been subject to a fair amount of comparative study in the urban studies literature (Garber and Imbroscio 1996; Reese 2006; Cullingworth 2015). In general, Canadian cities tend to be denser (Filion et al. 2004) and have more centrally focused employment markets (Garber and Imbroscio 1996; Filion et al. 2004; Reese and Rosenfeld 2004). Furthermore, government actors tend to play a more significant role in the real estate development process than their US counterparts (Garber and Imbroscio 1996).

Reese (2006) found that Canadian and American cities employ similar economic development strategies and policies in response to global economic trends and associated local fiscal challenges. Although there are differences in governance and federal arrangements for government structure between the two nations, “the organization of economic development (departmental structures and decision making processes) is essentially the same, economic stress is viewed in a similar fashion, and local officials define economic development goals in similar ways. And the same policies predominate” (Reese 2006, p. 556).

Though Reese (2006) does concede that planning happens differently in the two countries:

Planning is more accepted and more integral to policy in Canada. Perhaps the most fundamental difference is the strong American tradition of private property rights. The protection of these rights, dating from the foundation of the American nation in the 18th century, provides a significant check on the authority of government in general and planners in particular. In Canada the development of statutory and judicial protections of property rights came much later. As a result, Canadian planners enjoy greater latitude in pursuing the public good than do US planners (p. 556).

Regarding depopulation, research has established parallel patterns of depopulation in both the USA and Canada (Hall and Hall 2008; Warkentin 2012). The key difference, to some scholars, is the slow rate of decline in Canadian cities, in contrast to the more dramatic and faster decline more common in US cities (Hartt and Warkentin 2017; Warkentin 2012; Seasons 2007). Additionally, the tendency for Canadian cities to amalgamate with surrounding towns and suburbs has resulted in less population loss due to suburbanization. Nevertheless, research has explored depopulation in the Canadian context, as well as specific approaches to address the associated challenges (Hartt 2017a; Martinez-Fernandez et al. 2012a, b).

Turning to downtowns, research into retail business patterns has shown a discernable measure of sustained shrinkage in many Canadian Central Business Districts (CBDs). An examination of retail patterns in shrinking Canadian cities by the Center for the Study of Commercial Activity (CSCA) at Ryerson University found that “Growing cities have more stores in downtowns…” than declining cities (Simmons 2003, p. 6). The report went on to find that “while the commercial structure of a city is formed over a long period of time, and is unlikely to respond to population decline in the last five years, these results confirm the patterns observed when growth differences over 25 years were observed…” (p. 6).

Just as research in the USA has shown the impact of depopulation, Canadian research has confirmed that “the loss of population by a city may signal more than a simple decline in market size. The loss of population becomes a signal that both leads to disinvestment and discourages new investment and enterprises.” (p. 6). The cascade effect is the loss of even further commercial activity in downtowns, the focus of the next section.

### Literature on downtowns

Prior to discussing our research on downtowns, a brief introduction to the concept is necessary. Traditionally, downtowns have been the “regional hubs of commerce, culture, and shopping” (Morcol et al. 2008, p. 140). Defined more formally as a Central Business District (CBD), this zone is a place where public and private investment efforts are concentrated. The CBD is often used as a proxy to measure the overall health of a city’s broader real estate market. For example, several US Presidential Executive Orders have directed federal agencies to locate their major offices and courthouses in central business districts of cities (E.O. 12072 and E.O. 13006).

A lot of recent attention has been paid to what is described as a downtown renaissance in both growing and shrinking cities (Glaeser 2011). While many cities have been overwhelmed by millennials, new construction, and jobs, much more have been left behind. More common is a broader shift in technological, political, and economic forces that has pushed the center physically further and further outside these downtowns into regional shopping centers and suburban office parks (Lang 2003). This is a major challenge for a growing region as it sprawls far from its historic center, straining fiscal, transportation, and other infrastructure systems (Cullingworth 2015). The strain of sprawl presents unique, and arguably more onerous, challenges in a contemporary context of shrinking cities and regions.

Blakely and Leigh’s (2013) research found that during economic booms, downtowns benefit from an influx of residents and investments, spurning gentrification; but what about during downtimes? They, and others, have written about the role of government around the “provision of water and sewerage lines, street lighting, access roads, and sidewalks” (Blakely and Leigh 2013, p. 239). Overlay zoning allows developers to “place facilities where demand exists rather than where planners think they ought to be” (Blakely and Leigh 2013, p. 241). But it is Business Improvement Districts (BIDs), Tax Increment Financing, and public–private partnerships that have received the most attention as solutions to the problems of the shrinking downtown (Mallet 1994; Mitchell 2001; Mitchell 2008; Morcol et al. 2008). Blakely and Leigh (2013) conclude their research into reviving shrinking downtowns with this advice:

Many small towns believe tourism is the antidote to the collapse of their town centers. Although tourist dollars are important, local trade is important as well. Tourism is both cyclical and fickle. Commercial centers must ultimately survive on local traffic…One of the best ways to make the downtown area attractive is to embark on programs that give it character or restore some character to it. (p. 241)

What is missing in this literature is any substantial examination of ways that shrinking downtowns can adjust to a smaller business footprint, to fewer jobs, fewer businesses. A comparative view is especially useful here, because US downtowns have had a mixed history of efficacy in managing vacancy, while Canadian downtowns have been viewed as more successful:

For Canadians, downtowns have traditionally been regarded as safe, healthy, and vibrant places, especially when compared to their U.S. counterparts. However, multiple pressures from the suburbanization of population and commercial structure (facilitated by car-based transport infrastructure expansion), the general aging of the downtown, and the demise of the downtown department stores have resulted in the signs of decline in many of Canada’s downtowns (Morcol et al. 2008, p. 402).

### Mapping and measuring urban decline

Bowman and Pagano (2004) conducted an exhaustive study on vacant land seeking to understand the extent of the vacancy problem in the USA. They administered written surveys to local officials and assembled a database of abandoned buildings and vacant lot counts across more than 100 cities in the USA. Unfortunately, this survey-based method has been shown to be unreliable when crosschecked against housing unit counts from the Decennial Census. Local officials use very different strategies from one another to account for vacancy and abandonment, making the use of locally distinct administrative data sources a suboptimal approach for making national generalizations (Bowman and Pagano 2000). Hillier et al. (2003) examined Philadelphia’s housing databases to track vacancy and abandonment data, but their systems are not interoperable and study domain is of a limited scope, making broad-scope comparative analysis impossible. Wilson and Margulis (1994) developed a similarly localized analysis in Cleveland. Runfola and Hankins (2010) conducted field work in Atlanta to identify on-the-ground numbers of abandoned and derelict housing, but constructed information for only a limited subset of census block groups in the Atlanta region at a high time cost, making this approach infeasible for broad-scope analyses.

Many remote-sensing and GIS-based studies have worked toward measuring urban population change, including Weber and Puissant (2003), Xiao et al. (2006), and Yang and Lo (2002). Ryznar and Wagner (2001) attempted to use remote-sensing to study the effects of population decline. However, they were only able measure net changes in forested and agricultural land, and had to extrapolate their findings to examine housing and commercial land use changes. Banzhaf et al. (2007) explored shrinkage in Leipzig, Germany, but found that the necessary data to validate their findings was lacking.

In order to overcome these issues, recent work has utilized housing occupancy counts produced by the United States Postal Service (USPS), a novel database only recently made available to interested researchers (for a nominal fee). Though changes in housing occupancy can happen for a variety of reasons, these data correlated strongly with segments of the US Sunbelt that experienced widespread vacancy and abandonment during the Great Recession in the USA. Building on this system, the Canadian Census has recently made occupancy information available at census tract level, extrapolated based on broad-scope decennial surveys. Both of these approaches allow for the systematic collection and analysis of occupancy data at fine temporal resolutions^1^ and fill a gap in the literature to better understand the process of housing abandonment.

A bigger challenge is understanding abandonment in commercial centers and downtowns. USPS data are also available for commercial properties, but businesses do not constitute themselves in the same way that residential households do: there are minimum square foot requirements for domiciles, where a business can occupy an address with only a mailbox. Beyond government sources, real estate scholars have developed their own proprietary databases for measuring and studying commercial vacancy. The Centre for the Study of Commercial Activity at Ryerson University in Toronto regularly produces reports on the state of the commercial real estate industry in Canada. Hernandez et al. (2009) concluded that vacancy rates rose slightly in Greater Toronto between 2005 and 2009, based on data from shopping centers, power centers, and retail strips—1078 total retail venues. However, the data do not include the less formal commercial buildings typically found in historic centers and downtowns. In short, there is a lack of reliable, consistent data on the occupancy of downtown real estate in Canada and the USA. This deficiency of good data has limited our understanding of abandonment in commercial centers and downtowns.

## Conceptual framework

**Fig. 1:**
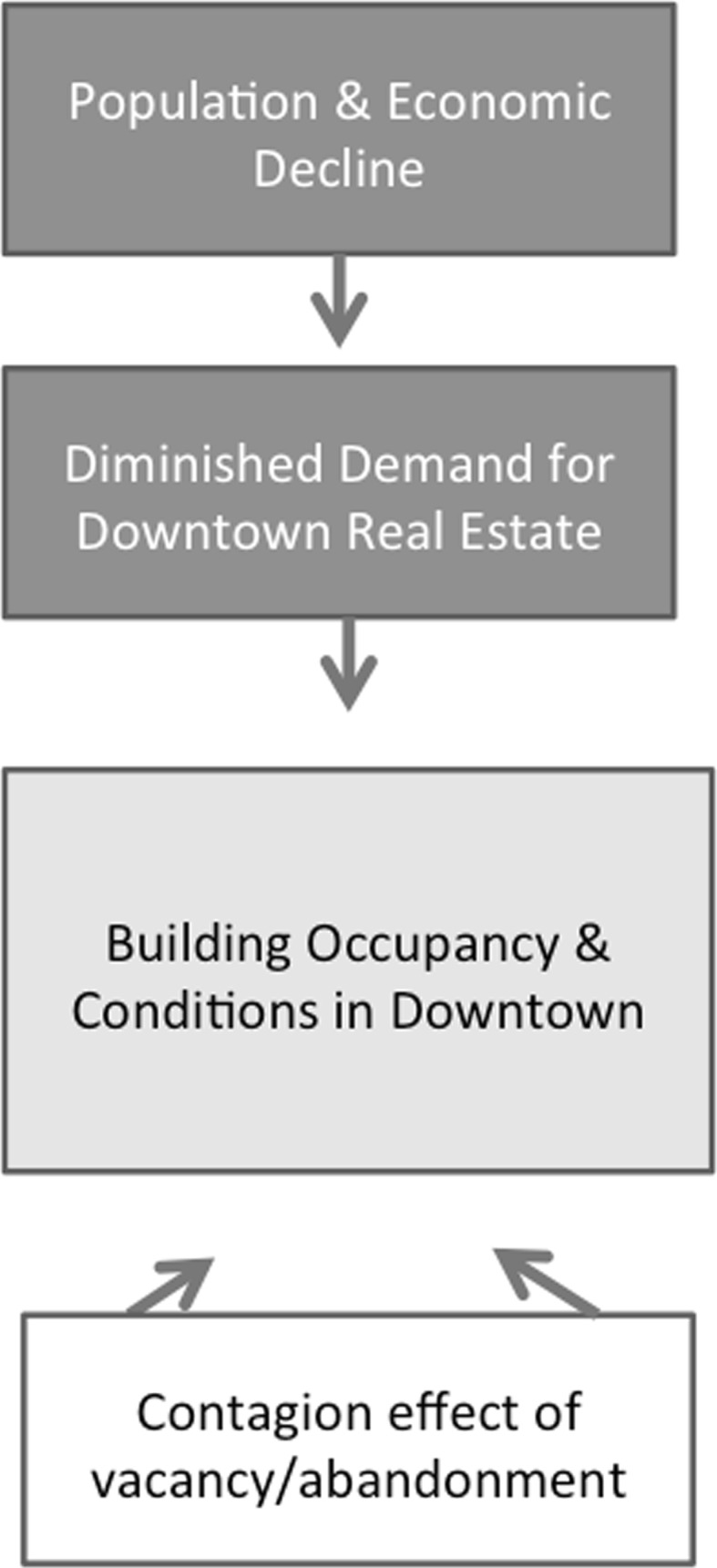
Conceptual framework of downtown decline

The aforementioned literature on shrinking cities and downtowns helps us consider a broader conceptual framework that can be applied in testing the following hypothesis: Differences in the process of urban decline in downtown districts vary due to national or historic context. While certainly the myriad of forces at work in cities and regions play a role in shaping what happens in a downtown, the scholarly literature cited above tells a story which we present in Fig. 1 below. Whatever broader economic, political, or social conditions might be at work, we begin at the top of the diagram with the notion that these forces will generate population and economic decline for some places (e.g., in a booming economy, there will be fewer of such declining places than in a weakening economy). But this paper seeks to isolate the direct and indirect impacts of that decline, rather than interrogating precisely why or how it is occurring.

What follows such decline will most always be a concomitant decrease in demand for downtown real estate. Given that a downtown functions as a center for a city, in the face of larger decline, the number of firms, organizations, investors, and individuals seeking real estate in the downtown is expected to fall. The consequence of such a drop in demand for downtown real estate is a decrease in the occupancy rates of downtown buildings. With lower rents (and lower profits), building conditions are also expected to degrade due to decreased investment in maintenance, protection, and care.

Unfortunately, the story does not end there due to a well-documented trend of how vacancy tends to spread (explored in the literature above). Figure 1 indicates how the economic and demographic forces push down from the top of the diagram and forces of vacancy and abandonment impact downtown building occupancy and conditions from below. Figure 1 depicts a potentially devastating spiral effect of decline, vacancy and abandonment, poor conditions, and depressed occupancy, contributing to a spreading effect of worsened conditions and occupancy. In this research, we seek to draw on this conceptual framework to examine how this process unfolds across two sister regions, noting the ways in which national planning systems might generate different outcomes.

## Methods

### Data

The empirical analyses run for this study focus on data sets that indicate change in downtown building stock. Population change is frequently used as a proxy to measure urban decline, though we find it to be an imperfect measure—and one that could in fact confound our analysis. Rather, this study looks to identify prevalence of increasing vacant commercial and residential spaces and buildings.

This study offers a methodology for cross-national comparison of downtown real estate trends over the last decade, adding to the gaps in the literature around the need to study, empirically, the processes of urban decline through a comparative lens. The choice of New England, Québec, and Ontario was made because they act as sister regions across an international border, with many common and many unique economic, political, and social frameworks and institutions, and their histories of managing vacancy and depopulation in their downtowns are regarded as quite different.

For New England, housing occupancy data aggregated and disseminated at the postal code level by USPS was utilized in conjunction with US Census demographic, housing, and employment data. For Québec and Ontario, all of the data are obtained from Canadian quinquennial Census collections from 2006 to 2011 at the census tract scale. Older data have been widely seen as incongruent with the 2006 data and no more recent data are available publicly. Likewise, only population data were publicly available at the municipality scale, making comparisons with the New England cities difficult.

To begin, we examined some basic demographic and housing characteristics of all New England, Ontario and Québec cities. Next, in order to more clearly understand the phenomenon of downtown shrinkage, we sought to develop a method to identify CBDs. From our list of all New England cities with a population over 20,000, we began to search online for zoning maps or Chamber of Commerce diagrams to indicate the boundaries of CBDs. We found 41 cities with delineated CBDs, and then were able to roughly map out the corresponding census tracts in each city (in most cities it was just one, but in some cases it was two tracts). To identify CBDs in Ontario and Québec, we used a combination of Google maps and Streetview and interviews with local officials to delineate boundaries.

Based on the theory that CBDs tend to be predominantly business oriented, we returned to the full list of New England cities and selected all census tracts within those cities. Next, we calculated the ratio of total businesses to total residences for the 16,000 census tracts that fulfill this designation. Among those 41 New England cities where we acquired precise CBD boundaries, the CBD census tracts ranked in the top 20% of the business-to-residence ratio, thus confirming the validity of this approach.

### Selection of cities and case study methodology

Case study cities were chosen from the results of the above analysis using a two-step process. First, cities with clear signs of population decline and downtown vacancy were identified. Of the remaining cities, selections were made with the aim of reaching a broad range of urban places, including differences in density, size, population levels, and economic conditions. Nine cities in total, three from New England, three from Ontario, and three from Québec, were sampled for the study.

New England is a diverse region, comprising six US states, Maine, New Hampshire, Vermont, Massachusetts, Rhode Island, and Connecticut. Its largest city is Boston and today boasts a mix of industrial, technological, service, and extractive industries. Ontario’s largest city is Toronto, which includes a substantial metropolitan area that dwarfs all other economic activity in the Ontario region, making Ontario that biggest contributor of all Canadian provinces toward GDP. Linguistically and culturally, Quebec is distinct from the rest of Canada and from New England, with its capital city of Montreal, and the region is a diverse mix of extractive, agricultural, high-tech, and industrial (Haddow 2015).

We conducted direct observation of the conditions of all buildings within the CBDs of the nine case study cities (Gaber and Gaber 2007). We developed a direct observation tool to record our observations. The unit of analysis we employed was the address. (So even if there are multiple units in a residential building or many different offices in a commercial building, we only looked at “4 Main Street” as one unit, for example.) The observation tool consists of three parts. First, identifying the “occupancy status”—a categorical variable ranging from 0 to 4 where 1 is fully occupied; 2 is partially occupied with “for rent” signs; 3 is unoccupied; 4 is illegally occupied with evidence of squatting; and 0 is for a location experiencing renovations/construction. Second, identifying the “structure condition”—again a categorical variable ranging from 0 to 4 where (1) is an empty lot where there is no structure or it is demolished; (2) is poor condition with evidence of fairly extensive disrepair, peeling paint or broken windows; (3) is fair condition with evidence of minor disrepair but is generally okay; (4) is excellent, in good condition, with exterior surfaces intact; and 0 is for a location experiencing renovations/construction. Lastly, we also took general notes to characterize the quality of the exterior of the building, or if the interior is notable.

We purposefully included key physical traits in the category descriptions (such as peeling paint for ‘2’ in the structure condition variable) to help minimize disparities in the data. However, the categorization of direct observations by multiple individuals inherently introduces some subjectivity and potential inconsistencies to the data collection process. To further limit direct observation variance, if any team member was unsure of how to score a given address during a visit, other members of the research team were solicited for their opinions either based upon photographs taken or based on Google maps street view. Likewise, if there were buildings that did not have clear addresses during the visit, we sought to determine this with online mapping sites (or just by typing a business name into a search engine). This not only avoided incorrect categorizations, the discussion and analysis helped build understanding and cohesion within the research team. Due to limitations in our travel budget, we did not physically visit Baie-Comeau, instead relying on interviews with local officials and conducting a Google Streetview tour of the CBD there.

### Analysis

In order to analyze the direct observation data, we first mapped the spatial patterns of occupancy and condition. The data were geocoded using tabular data and Google’s geocoding engine. The resulting KML file was imported to ArcGIS and joined with the complete qualitative findings. Inverse distance weighted interpolation was used with a fixed search radius of 0.001 to complete the dataset. Lastly, we adjusted the symbology of the results from the inverse distance weighted interpolation to create the heat map visualizations.

We also conducted spatial analysis of the direct observation data using GeoDa software to compliment the visual analysis of the mapped data. In order to capture whether any of the cities exhibited statistically significant clustered patterns of occupancy or condition, the spatial autocorrelation of the direct observation data was tested by calculating global Moran’s I as an overall measure of the phenomenon and Local Indicator of Spatial Association (LISA) to gauge the local effects of spatial autocorrelation. Moran’s I (Moran 1950) tests the null hypothesis that the variable being examined is randomly distributed. If a significant *p* value is returned, the null hypothesis is rejected meaning that either: (1) the spatial distribution of high and/or low points are more spatially clustered than would be expected (indicated by a positive z-score), or (2) the spatial distribution of high and low points are more spatially dispersed than would be expected (indicated by a negative z-score). Although Moran’s I can tell us whether spatial autocorrelation occurs, it does not provide detailed evidence of where statistically significant clusters occur. LISA provides a more localized analysis by assessing the similarity of each observation with its surroundings. LISA analysis detects five potential scenarios: (1) hot spots (locations with high values and high similarity with surroundings), (2) cold spots (locations with low values and high similarity with surroundings), (3) spatial outliers (locations with high values and low similarity with surroundings, (4) spatial outliers (locations with low values and high similarity with surroundings), and (5) locations without statistically significant autocorrelations.

The concept of spatial autocorrelation relies on notion that contiguous spatial elements can influence one another. Generally, a dichotomic contiguity matrix is defined based on whether one spatial area touches the boundary of another. However, with point data, a threshold distance is defined to gauge whether two points are contiguous. As this analysis concentrates on downtown CBDs, we defined the threshold distance to be 243 m (800 feet)—roughly two average North American city blocks. And since the observation data was categorical, dummy variables were created to test the spatial autocorrelation of each of the occupancy and condition variables (0 through 4) individually, as well as select combinations.

## Results

### Descriptions and analysis of demographic data

The US Census, combined with other US real estate and housing databases, allowed for a rich exploration of both historic trends and contemporary conditions in New England at a fairly fine-grained level. In contrast, the Canadian Census underwent a major transformation recently, making historical comparisons difficult and the limitations of publicly available data make the results quite coarse geographically.

For New England, we began with a broad picture of all 182 cities and towns with a population of 20,000 or higher in the 2010 US Decennial Census. Among those cities, their populations grew on average 17% between 1980 and 2010, and their number of occupied housing units grew by 31%. But growth was not a hallmark of all New England cities (see Table 1). During this same time period, 24 lost more than 2% of their population and six lost occupied housing units (cities that lost occupied housing units included: Newport [RI], Hartford [CT], Holyoke [MA], Woonsocket [RI], Bridgeport [CT], New Britain [CT], New London [MA], Belmont [MA], and Newport [RI]).

**Table 1:** Population decline analysis of cities in New England

State	No. cities in study	Stable cities (pop change of < 2% ±)	Growing cities (pop growth > 2%)	Shrinking cities (pop decline > 2%)	No. cities increased OHU	No. cities decreased OHU	No. cities where pop and OHU increased	No. Cities where pop and OHU decreased
CT	52	3	45	4	47	2	45	2
ME	7	1	5	1	6	0	5	0
MA	91	9	68	14	80	2	68	2
Nh	13	0	12	1	13	0	12	0
RI	18	1	13	4	15	2	13	2
VT	1	0	1	0	1	0	1	0
Total	182	14	144	24	162	6	144	6

*OHU* occupied housing units

For Canada, we started by looking at all 72 cities in Ontario and Québec. However, due to data limitations, we only had access to data over a much shorter time period (the 2006 and 2011 censuses). Following the Canadian Census definitions, dwellings occupied by usual residents was used as a proxy for occupied housing units. The results show in Table 2, that like New England, many Ontario and Québec cities and towns are likewise experiencing depopulation. Among the 29 Québec cities, three lost more than 2% and six were roughly stable. Among the 43 Ontario cities, one lost more than 2% and 24 were stable. Given all of the municipal consolidation in both regions in recent years, it is quite remarkable that the Canadian Census would show any net population decline, let alone more than 2% in just 5 years.

**Table 2:** Population decline analysis of cities in Ontario and Québec

Province	No. cities in study	Stable cities (pop change of < 2% ±)	Growing cities (pop growth > 2%)	Shrinking cities (pop decline > 2%)	No. cities increased DOUR	No. cities decreased DOUR	No. cities where pop and DOUR increased	No. cities where pop and DOUR decreased
Ontario	43	24	18	1	41	2	18	1
Quebec	29	6	20	3	25	4	19	2
Total	72	30	38	4	66	6	37	3

*DOUR* dwellings occupied by usual residents

Based on the results from our quantitative analysis, nine cities from the three regions were selected as individual case studies. Cities were selected in each region where sustained population, housing, and economic decline were present and they were, statistically speaking, representative of shrinking cities in their region across density, size, population levels, and economic conditions. Additionally, in our selection, we sought some degree of geographic diversity. Bridgeport (CT), Springfield (MA), and Lewiston (ME) were selected from New England. Leamington, Windsor, and Chatham-Kent were selected from Ontario. And Shawinigan, Baie-Comeau, and Dolbeau-Mistassini were selected from Québec. Table 3 provides descriptive statistics for each of the case study cities.

**Table 3:** Descriptive statistics for case study cities

City	Population	Population change (%)	OHUs	OHU change (%)
Bridgeport, CT	144229	1.18	51255	− 1.48
Springfield, MA	153060	0.49	56752	2.57
Lewiston, ME	36592	− 9.61	15267	2.05
Leamington, ON	49765	0.05	17735	1.72
Windsor, ON	319246	− 1.27	127025	0.81
Chatham-Kent, ON	104075	− 4.16	43095	− 1.63
Shawinigan, QC	55009	− 2.48	26335	0.61
Baie-Comeau, QC	28789	− 2.98	12565	− 0.59
Dolbeau-Mistassini, QC	16019	− 1.46	7020	9.77

(1) Population estimates for US cities from 2010 US census; for Canadian cities from 2011 Canadian census

(2) Population change for US cities between 1980 and 2010; for Canadian cities between 2006 and 2011

(3) Dwellings occupied by usual residence collected from Canadian census as proxy for occupied housing units

(4) Change in OHUs for US cities between 1980 and 2010, for Canadian cities between 2006 and 2011

### Direct observation data

For each CBD we collected between 62 (Baie-Comeau) and 270 (Shawinigan) observations (see Appendix 1). While not the total universe of all addresses in each CBD, these observations accounted for most of the addresses in the most important, central business areas of each city.

For the Ontario cities, Windsor was in the worst shape regarding occupancy, with 28% of all addresses showing either partial or complete vacancy. Leamington and Chatham-Kent had 15 and 20% partial or complete vacancy, respectively. Regarding condition, Windsor also did the worst, with five properties rated as poor, where neither Leamington nor Chatham-Kent registered any as poor.

These figures are significantly better than what emerged in the New England cities, where more than one-third of Springfield’s addresses were either partially or fully vacant and Lewiston and Bridgeport also reporting more than one-quarter of such properties. Likewise, the New England cities have proportionally much lower quality building conditions in their downtowns, ranging from 3 to 4% getting a poor rating.

Turning to Québec, Shawinigan stands out as having 22% vacant addresses, but the much smaller Dolbeau-Mistassini and Baie-Comeau only showing 8 and 4% vacancy, respectively. The same trend held true for building conditions, where Shawinigan had eight properties rated as poor (3%) where the other two cities had none rated as poor.

**Fig. 2:**
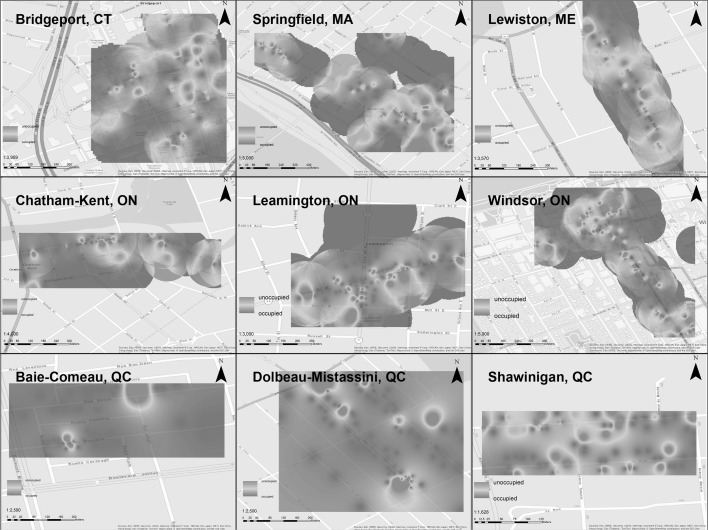
Heat map analysis of occupancy of downtown real estate in all nine case studies

**Fig. 3:**
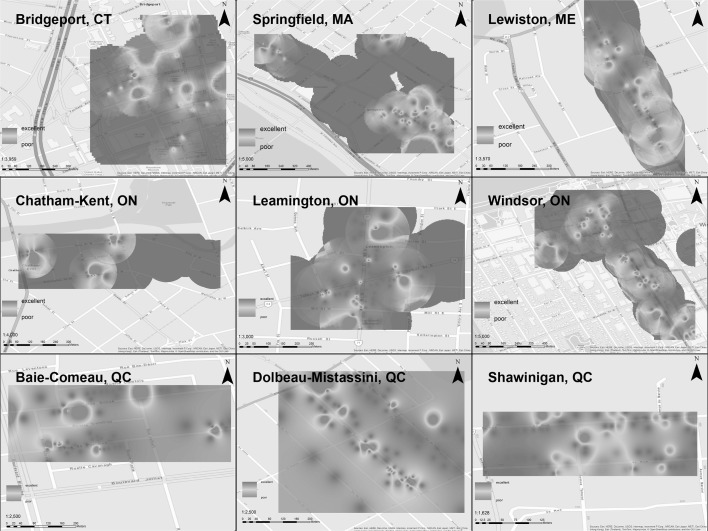
Heat map analysis of condition of downtown real estate in all nine case studies

To better understand these results and their spatial distribution, we mapped and analyzed these trends for all nine cities. The maps show where concentrations of vacancy (Fig. 2) and poor conditions (Fig. 3) tend to exist, the intensity and strength of those patterns. A comparative look across these regions shows a discernably stronger scope and scale of vacancy in the New England and Ontario downtown, as compared to the Québec cities. Of particular interest is the tendency for spatial clustering in the New England cities, and to a lesser extent the Ontario cities. For both Bridgeport and Windsor, a clustering in the northern edge of the CBDs suggests a major source of disinvestment in that area, generating above average vacancy levels. This contrasts with the Shawinigan, which has comparable vacancy levels, but where there is no cluster or hot spot of vacancy.

Examining the condition of the CBDs (Fig. 3), the New England and Ontario cities once again demonstrate the spatial polarization of their downtown built environment. Pockets of poor and excellent condition areas are clearly visible in the maps. In many of the cities (Bridgeport, Springfield, Lewiston), the spatial clustering of poor condition built environment closely matches the spatial clustering of vacancy. However, in Windsor and Chatham-Kent there are distinctly different patterns of condition and occupancy. In complete contrast, the three Québec cities show a relatively even distribution of poor condition real estate in the CBD.

### Spatial analysis of direct observation data

The spatial autocorrelation of the direct observation data was first measured using global Moran’s I. Results from the spatial analysis of the direct observation data echo the trends identified visually using the heat maps (Figs. 2, 3). Concentrating on trends of poor condition and low occupancy, we found that there was no statistically significant clustering of empty lots or buildings in poor condition (condition variables 1 and 2, respectively). And although there were practically no observations of illegal occupation in any of the cities (occupancy variable 4), there were statistically significant clustering patterns of partially occupied and unoccupied buildings in several cities (occupancy variables 2 and 3, respectively). Table 4 details select results from the spatial analysis. (Full results are given in Appendix 2.)

The stronger scope and scale of vacancy in New England and Ontario inferred from the heat map analysis was confirmed as Bridgeport, Lewiston, Leamington, and Windsor all had statistically significant clustering patterns. In Lewiston, partially occupied buildings with for rent signs (occupancy variable 2) were clustered, whereas the other three cities had clusters of unoccupied buildings (occupancy variable 3). As global Moran’s I only provides insight to the study area as a whole, the significant results in Bridgeport, Lewiston, Leamington, and Windsor encourage further local analysis.

**Fig. 4:**
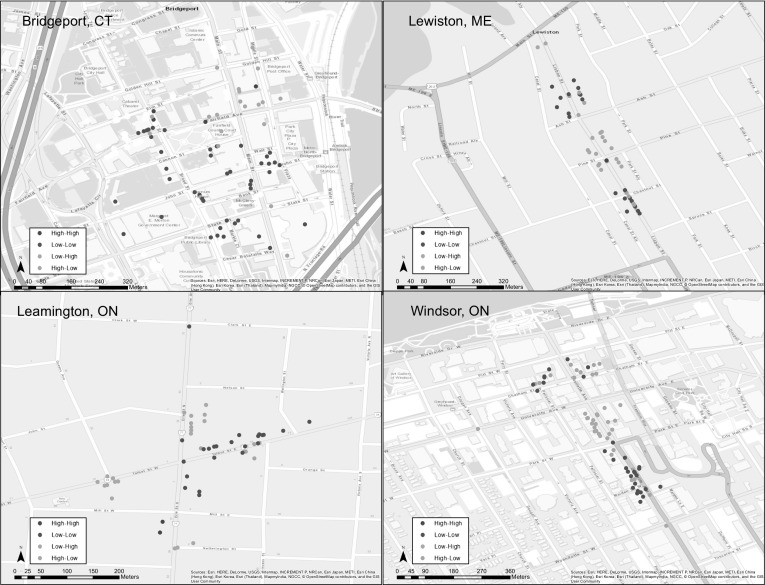
LISA cluster map of occupation in Bridgeport, Lewiston, Leamington and Windsor, where high–high depicts hot spots of vacancy

Results from LISA (Fig. 4) depict the centers of “hot” and “cold” spots as well as spatial outliers (points that were not statistically significant are not included in the figures). As we saw in the occupancy heat map of Bridgeport, Fig. 4 demonstrates that clusters of low occupancy (red dots) in Bridgeport are all located at the northern edge of the CBD. In contrast, the lone statistically significant cluster of low occupancy in Lewiston is squarely in the middle of downtown. Like Bridgeport, clusters of low occupancy in Leamington are also at the edge of downtown; however, in this case they are to the south. Lastly, Windsor had no “hot spots” of low occupancy, only “cold spots”—especially in the southern half of the downtown area (Fig. 4).

**Table 4:** Spatial analysis of occupancy in nine case study cities

City	Occupancy 2		Occupancy 3	
	*z-*score	*p* value	*z-*score	*p* value
Bridgeport, CT	0.542	0.588	5.938	0***
Springfield, MA	0.719	0.472	1.434	0.152
Lewiston, ME	2.586	0.0097***	7.73	0.465
Leamington, ON	–	–	2.101	0.0356**
Windsor, ON	0.205	0.837	2.789	0.00529***
Chatham-Kent, ON	− 0.569	0.569	0.191	0.849
Shawinigan, QC	1.544	0.122	− 0.248	0.804
Baie-Comeau, QC	0.957	0.339	–	–
Dolbeau-Mistassini, QC	− 0.454	0.65	–	–

***Denotes significance at 0.01 level

**Denotes significance at 0.05 level

## Discussion and conclusions

With measurable population decline and increases in vacancy across Ontario, Québec, and New England, what we sought to do in this research was to understand how that change manifested spatially in downtown areas. Drawing on a conceptual framework presented in Fig. 1, we were able to track the forces of decline in both regions and pick up on evidence of a spreading effect in some cities, but ultimately discovered varying impacts of decline on occupancy and building conditions. We found that while the scale of population decline was certainly greatest in New England, both Ontario and Québec have seen substantial decline and have appeared to have better weathered the change. This may also be due, in part, to the slower pace of change in the Canadian provinces, relative to the New England cities.

The Québec cities show less vacancy and less concentration of poor condition buildings. Baie-Comeau and Dolbeau-Mistassini, in particular, appeared to have managed depopulation and adjusted to a smaller population better than the New England cities. In Ontario, Windsor looked most similar to the New England cities, with high levels of vacancy and relatively poor building conditions, where the smaller Ontario cities looked more like their Québec counterparts. In general, the smaller cities faired better than large ones. (Baie-Comeau, Dolbeau-Mistassini, and Leamington all have populations under 50,000, while Windsor has 319,246 residents and Chatham-Kent has 104,075.) This could be the result of less relative activity and dependence on the downtown area due to a more rural economic base. The downtowns of these smaller cities simply may not play the same role as their bigger city equivalents and, therefore, have not experienced the same physical decline.

The research also raises key questions about the differences between residential and commercial land use in city centers. We focused here on commercial uses, but residential patterns may also play a role in occupancy and conditions of buildings. Future research should seek to further untangle the relationships between residential and commercial land uses in these shrinking downtowns. The spatial analysis revealed clusters of low occupancy in several of the cities. The direct observation data and resulting heat and LISA maps (Figs. 2, 3, and 4) show a distinctive pattern of hot spots of vacancy in the edges of the downtowns. Local officials can use this information to develop land use plans for those areas, considering market demand and the overall health of the local and regional economy. Specifically, these edge zones may be better suited for non-CBD uses, in order to direct and focus CBD uses (office, retail, housing, arts) into what could then be a smaller, denser CBD.

All three of the Québec cities examined had no statistically significant clusters of low occupancy. Cities, such as Shawinigan, with no identified vacancy hot spots demonstrate an opportunity to rethink the core of the CBD. Here, a new thinking around future uses can be valuable. While much of the shrinking cities literature has examined the programming of shrinking residential areas for non-residential uses, the shrinking downtown is harder to envision that way. In this research, we have found that many of the shrinking cities studied are surrounded by rich and economically vital agricultural regions. While urban agriculture has been widely seen as a prospective future use in residential areas of shrinking cities, CBDs would hardly seem to be compatible with agriculture. But with proper siting and considerations for preserving existing streetscapes, it might not be such a crazy idea.

We recommend downtown planners consider the emerging urban practice of hydroponic, indoor, (also known as vertical) farming. Using containers retrofitted to support hydroponic farms, planners can work with property owners to preserve the historic facades and exteriors of CBD buildings and then “drop” in these pre-built, pre-configured, and module hydroponic containers. Efforts to do this in relatively open areas of Boston have been met with much success, and we recommend exploration of this idea for the shrinking downtowns which have been the subject of this study (Abraham 2015; National Public Radio 2015).

## Appendix 1

**Table:**

Windsor	Count 0 s	Count 1 s	Count 2 s	Count 3 s	Count 4 s	Count ?s	Total count
Occupation	0	122	16	32	0	1	171
Condition	0	1	5	28	137	0	171

Windsor	% 0 s	% 1 s	% 2 s	% 3 s	% 4 s	% ?s	Total %
Occupation	0.00	71.35	9.36	18.71	0.00	0.58	100.00
Condition	0.00	0.58	2.92	16.37	80.12	0.00	100.00

Leamington	Count 0 s	Count 1 s	Count 2 s	Count 3 s	Count 4 s	Count ?s	Total count
Occupation	0	120	2	20	0	3	145
Condition	0	3	0	15	127	0	145

Leamington	% 0 s	% 1 s	% 2 s	% 3 s	% 4 s	% ?s	Total %
Occupation	0.00	82.76	1.38	13.79	0.00	2.07	100.00
Condition	0.00	2.07	0.00	10.34	87.59	0.00	100.00

Chatham-Kent	Count 0 s	Count 1 s	Count 2 s	Count 3 s	Count 4 s	Count ?s	Total Count
Occupation	1	86	6	18	0	8	119
Condition	1	0	0	5	113	0	119

Chatham-Kent	% 0 s	% 1 s	% 2 s	% 3 s	% 4 s	% ?s	Total %
Occupation	0.84	72.27	5.04	15.13	0.00	6.72	100.00
Condition	0.84	0.00	0.00	4.20	94.96	0.00	100.00

Springfield	Count 0 s	Count 1 s	Count 2 s	Count 3 s	Count 4 s	Count ?s	Total Count
Occupation	2	93	24	30	0	1	150
Condition	2	3	5	25	114	1	150

Springfield	% 0 s	% 1 s	% 2 s	% 3 s	% 4 s	% ?s	Total %
Occupation	1.33	62.00	16.00	20.00	0.00	0.67	100.00
Condition	1.33	2.00	3.33	16.67	76.00	0.67	100.00

Lewiston	Count 0 s	Count 1 s	Count 2 s	Count 3 s	Count 4 s	Count ?s	Total Count
Occupation	1	78	16	11	1	2	109
Condition	1	4	5	18	81	0	109

Lewiston	% 0 s	% 1 s	% 2 s	% 3 s	% 4 s	% ?s	Total %
Occupation	0.92	71.56	14.68	10.09	0.92	1.83	100.00
Condition	0.92	3.67	4.59	16.51	74.31	0.00	100.00

Bridgeport	Count 0 s	Count 1 s	Count 2 s	Count 3 s	Count 4 s	Count ?s	Total Count
Occupation	8	109	14	38	1	3	173
Condition	8	8	6	28	122	1	173

Bridgeport	% 0 s	% 1 s	% 2 s	% 3 s	% 4 s	% ?s	Total %
Occupation	4.62	63.01	8.09	21.97	0.58	1.73	100.00
Condition	4.62	4.62	3.47	16.18	70.52	0.58	100.00

Shawinigan	Count 0 s	Count 1 s	Count 2 s	Count 3 s	Count 4 s	Count ?s	Total Count
Occupation	1	209	39	21	0	0	270
Condition	1	2	8	52	207	0	270

Shawinigan	% 0 s	% 1 s	% 2 s	% 3 s	% 4 s	% ?s	Total %
Occupation	0.37	77.41	14.44	7.78	0.00	0.00	100.00
Condition	0.37	0.74	2.96	19.26	76.67	0.00	100.00

Dolbeau-Mistassini	Count 0 s	Count 1 s	Count 2 s	Count 3 s	Count 4 s	Count ?s	Total Count
Occupation	0	107	7	2	0	0	116
Condition	0	1	0	15	100	0	116

Dolbeau-Mistassini	% 0 s	% 1 s	% 2 s	% 3 s	% 4 s	% ?s	Total %
Occupation	0.00	92.24	6.03	1.72	0.00	0.00	100.00
Condition	0.00	0.86	0.00	12.93	86.21	0.00	100.00

Baie-Comeau	Count 0 s	Count 1 s	Count 2 s	Count 3 s	Count 4 s	Count ?s	Total Count
Occupation	0	59	2	1	0	0	62
Condition	0	0	0	9	53	0	62

Baie-Comeau	% 0 s	% 1 s	% 2 s	% 3 s	% 4 s	% ?s	Total %
Occupation	0.00	95.16	3.23	1.61	0.00	0.00	100.00
Condition	0.00	0.00	0.00	14.52	85.48	0.00	100.00

## Appendix 2

**Table:**

		Bridgeport	Springfield	Lewiston	Leamington	Windsor	Chatham-Kent	Shawinigan	Baie-Comeau	Dolbeau-Mistassini
CO	*z*-score	0.9913	–	–	–	–	–	–	–	–
	*p* value	0.3216	–	–	–	–	–	–	–	–
C1	*z*-score	− 0.0401	–	0.0849	–	–	–	–	–	–
	*p* value	0.9680	–	0.9324	–	–	–	–	–	–
C2	*z*-score	–	1.3093	0.1134	–	0.9726	–	0.3470	–	–
	*p* value	–	0.1904	0.9097	–	0.3308	–	0.7286	–	–
C3	*z*-score	2.5127	− 0.9600	1.2041	4.3617	3.7167	− 0.1394	1.6465	− 0.3322	0.4773
	*p* value	0.0120	0.3371	0.2285	0.0000	0.0002	0.8891	0.0997	0.7398	0.6331
C4	*z*-score	4.0366	− 0.0445	1.3707	3.7317	3.8193	− 0.1394	1.7833	− 0.3322	0.4107
	*p* value	0.0001	0.9645	0.1705	0.0002	0.0001	0.8891	0.0745	0.7398	0.6813
C1&2	*z*-score	− 0.6072	0.9938	0.7395	0.2479	0.6458	–	0.3470	–	–
	*p* value	0.5437	0.3203	0.4596	0.8042	0.5184	–	0.7286	–	–
O0	*z*-score	0.9913	–	–	–	–	–	–	–	–
	*p* value	0.3216	–	–	–	–	–	–	–	–
O1	*z*-score	2.5134	0.3282	1.2576	1.8359	1.1876	− 1.2825	0.2604	0.5591	− 0.3258
	*p* value	0.0120	0.7428	0.2085	0.0664	0.2350	0.1997	0.7946	0.5761	0.7446
O2	*z*-score	0.5421	0.7191	2.5863	–	0.2051	− 0.5689	1.5444	0.9566	− 0.4538
	*p* value	0.5877	0.4721	0.0097	–	0.8375	0.5694	0.1225	0.3387	0.6500
O3	*z*-score	5.9381	1.4337	0.7300	2.1011	2.7887	0.1910	− 0.2479	–	–
	*p* value	0.0000	0.1517	0.4654	0.0356	0.0053	0.8486	0.8042	–	–
O4	*z*-score	–	–	–	–	–	–	–	–	–
	*p* value	–	–	–	–	–	–	–	–	–
O3&4	*z*-score	5.8343	1.4337	0.4170	2.1011	2.7887	0.1910	− 0.2479	–	–
	*p* value	0.0000	0.0152	0.6767	0.0356	0.0053	0.8486	0.8042	–	–
O2&3	*z*-score	2.8724	0.3752	1.8357	1.8359	1.1876	− 1.2825	0.2604	0.5591	− 0.3258
	*p* value	0.0041	0.7075	0.0664	0.0664	0.2350	0.1997	0.7946	0.5761	0.7446

## Footnotes

1 To date, no external validation of these survey-based approaches has been conducted by the academic community, and could provide a fruitful next-step for this style of research. Additionally, the authors agree that an accuracy assessment of this data could be very powerful, but also note that the USPS and Canadian Census conduct a great deal of accuracy internally before releasing the information. Where assessment could improve our understanding is in providing insight on how the timing of data collection might influence these types of analyses, which is a research question in and of itself.
